# Temporal Effects of Tranexamic Acid Administration on Functional Endoscopic Sinus Surgery

**DOI:** 10.7759/cureus.92893

**Published:** 2025-09-22

**Authors:** Tomotaka Hemmi, Kazuhiro Nomura, Rei Mashiko, Shinkichi Morita, Mitsuru Sugawara

**Affiliations:** 1 Otolaryngology, Japanese Red Cross Ishinomaki Hospital, Ishinomaki, JPN; 2 Otolaryngology, Tohoku Kosai Hospital, Sendai, JPN

**Keywords:** bleeding, endoscopic score, functional endoscopic sinus surgery, operative time, tranexamic acid

## Abstract

Introduction

Functional endoscopic sinus surgery (FESS) is a commonly performed procedure, and tranexamic acid (TXA) has been shown to improve surgical outcomes. However, no previous studies have examined the time-dependent effects of TXA within the same patient during surgery. This study was conducted to address this gap.

Methods

This study included patients who underwent bilateral full-house FESS performed by a single surgeon at each institution. Patient demographics, operative times, and endoscopic scores for each side were retrospectively collected and analyzed. A within-patient design was used by comparing the first-operated side with the second-operated side during bilateral FESS.

Results

A total of 51 patients were included. The mean operative times for the first and second sides were 28 min 25 s and 28 min 49 s, respectively. The mean endoscopic scores were 1.4 and 1.5, respectively; these differences were not statistically significant. Patients were further stratified into eosinophilic chronic rhinosinusitis (ECRS) and non-ECRS groups to evaluate differences in operative time and endoscopic scores. No significant differences were found between the two groups.

Conclusion

Intravenous administration of 1000 mg TXA before surgery allowed bilateral full-house FESS to be performed with comparable quality on both sides. If the procedure is completed within the approximate 2-hour elimination half-life of TXA, additional or continuous administration may not be necessary.

## Introduction

Functional endoscopic sinus surgery (FESS) is a widely adopted minimally invasive procedure aimed at improving the sinonasal function. Endoscopic surgery performed via transnasal insertion offers a minimally invasive alternative to traditional external approaches. This technique not only benefits patients by reducing surgical invasiveness but also provides surgeons with improved visualization, allowing for more delicate and precise manipulation. Moreover, the ability to share intraoperative findings in real time with other medical staff has significantly contributed to collaborative decision-making. These advantages underscore the substantial impact of endoscopic surgery on clinical practice, reflecting remarkable advancements in surgical technology. However, it still carries the risk of serious complications such as those involving the orbital and cranial region [1]. Effective control of intraoperative bleeding and securing a clear surgical field are crucial for ensuring surgical safety [2]. Tranexamic acid (TXA), a synthetic analogue of lysine, functions as an antifibrinolytic agent by competitively inhibiting the lysine-binding sites on plasminogen. This inhibition prevents the association of plasminogen with plasmin and fibrin, thereby suppressing fibrinolysis. Consequently, the stabilization of the fibrin matrix formed during secondary hemostasis is achieved through reduced plasminogen activation [3]. TXA has been reported to reduce intraoperative blood loss, improve surgical field visibility, and shorten the operative time in both FESS and endoscopic skull base surgery, with its clinical efficacy being well established [2,4]. Nevertheless, to the best of our knowledge, no study has evaluated the time-dependent effects of TXA administration in the same patient. Therefore, in this study, we employed a within-patient design by comparing the first-operated side with the second-operated side during bilateral FESS, aiming to investigate whether the effects of TXA on surgical outcomes vary over time in patients undergoing bilateral full-house FESS.

## Materials and methods

Subjects

This retrospective study was conducted following the Strengthening the Reporting of Observational Studies in Epidemiology (STROBE) guidelines, except for justifying the sample size [5]. We evaluated patients who underwent bilateral full-house FESS at the Otolaryngology Department of the Japanese Red Cross Ishinomaki Hospital and Tohoku Kosai Hospital in Japan between October 2024 and April 2025. Data regarding age, sex, presence of bronchial asthma, peripheral blood eosinophil percentage, mucosal eosinophil count, operative procedures, operative time, intraoperative blood loss, and surgical field quality were extracted from the medical records and retrospectively reviewed by a single independent assessor who was blinded to patient information. Surgical field quality was evaluated using the five-point scores by Boezaart et al. [6]. Nine patients with a history of surgery or those for whom pathologies differed between the left and right paranasal sinuses were excluded.

This study was approved by the Ethics Committee of Japanese Red Cross Ishinomaki Hospital and Tohoku Kosai Hospital (24-25, kkrtohoku-202503otor1_S1-01) and complied with the ethical standards as laid down in the 1964 Declaration of Helsinki and its later amendments or comparable ethical standards. Given the retrospective nature of this study, individual informed consent was not required. Instead, information regarding the study was disclosed on the hospital's website, and patients were allowed to opt out if they so desired.

Surgical procedure

Each procedure was performed under general anesthesia, using either inhalational anesthesia or total intravenous anesthesia, by two board-certified otorhinolaryngologists (T. H. and K. N.). In all cases, surgery was performed in the reverse Trendelenburg position, with the head elevated by 15°. Preoperative steroid administration and intraoperative dexmedetomidine infusion were not performed in any of the cases.

At the start of the surgery, 1000 mg TXA was administered intravenously. When necessary, septoplasty, including the correction of caudal deviation and complete removal of intranasal polyps, was performed before FESS. Before initiating FESS, 1% lidocaine with 1:100000 epinephrine was injected at the superior attachment and the posterior-inferior end of the middle turbinate. As a general rule, FESS was started on the right side; however, in cases where septal deviation made it difficult to ensure a sufficient interval after the administration of TXA, FESS was performed on the left side first.

During FESS, the uncinate process and anterior ethmoidal cells were completely removed, and both the frontal and maxillary sinuses were maximally opened into the nasal cavity. The surgical field was scored immediately before the removal of the ground lamella. Subsequently, the posterior ethmoid cells were completely removed, and the sphenoid sinus was opened. The sphenoid sinus was maximally opened toward both the olfactory and ethmoid sinuses. The middle and superior turbinates were preserved in all cases. The start of FESS was defined as the initiation of uncinectomy, and the end was defined as the point at which all paranasal sinuses were unified into a single cavity and no further removable septations or lesions remained. The duration of full-house FESS on each side was recorded accordingly. In patients for whom it was deemed necessary, inferior turbinate reduction and posterior nasal neurotomy were performed following completion of bilateral full-house FESS. These standardized protocols were applied to all cases.

Statistical analyses

For descriptive statistics, values were presented as the mean ± standard deviation. A paired t-test or the Mann-Whitney U test was used to analyze continuous variables (operative time on the left and right sides). Categorical variables (endoscopic scores) were analyzed using the Wilcoxon signed-rank test or the Mann-Whitney U test. Before performing parametric tests, the Kolmogorov-Smirnov test was conducted to confirm that the data followed a normal distribution. Statistical significance was defined as a p-value < 0.05. The test statistics and 95% CIs were also calculated. All statistical analyses were performed using EZR ver. 1.68, which is a modified version of the R Commander designed to add statistical functions frequently used in biostatistics [7]. The statistical power was not calculated. Given the within-subject design, where both sides of the sinonasal cavity in the same individual were compared, major demographic and clinical confounders were inherently controlled. Nevertheless, subgroup analyses were conducted by stratifying patients into two phenotypically distinct groups to assess potential interaction effects.

## Results

Subjects’ characteristics

A total of 51 patients underwent bilateral full-house FESS during the study. The demographic data of the patients are presented in Table 1. Approximately half of the patients had comorbid bronchial asthma, and eosinophilic infiltration of the nasal tissue meeting the diagnostic criteria for eosinophilic chronic rhinosinusitis (ECRS) was observed in approximately 45% of cases. In most cases, inferior turbinate reduction and septoplasty were performed in addition to full-house FESS. The mean total operative time, including bilateral FESS, additional surgical procedures, irrigation, hemostasis, suturing, and nasal packing, was 136.1 min.

**Table 1:** Patients’ information, diagnosis, and operative procedure. The diagnosis and operative procedures are indicated with duplicates. HPF: high-power field.

Item	Value
Patients (n)	51
Age (years)	54.0 ± 14.8
Male/female	29/22
Peripheral blood eosinophil (%)	5.9 ± 4.4
Mucosal eosinophil ≥ 70 per HPF	23/51
Presence of bronchial asthma Y/N	25/26
Operative procedure (number of operations)	
Functional endoscopic sinus surgery	51
Inferior turbinectomy	47
Septoplasty	43
Posterior nasal neurotomy	4
Total operative time (min)	136.1 ± 28.7
Intraoperative blood loss (mL)	46.4 ± 37.2

Temporal effects of TXA administration

In approximately 70% of the cases, FESS was first performed on the right side. The mean operative time was 28 min 25 s on the first side of the operation and 28 min 49 s on the second side. Although there was a slight increase in the operative time on the second side, the difference was not statistically significant. The mean endoscopic scores were 1.4 on the first side and 1.5 on the second side; however, this difference was not statistically significant (Table 2). Additionally, we analyzed changes in operative time and endoscopic scores by dividing the subjects into the ECRS and non-ECRS groups. Although the ECRS group showed a slightly longer operative time and a greater increase in endoscopic scores than the non-ECRS group, these differences were not statistically significant (Table 3). No adverse events related to TXA administration such as thrombosis or surgical complications were observed.

**Table 2:** Comparison of operative time and endoscopic score. FESS: functional endoscopic sinus surgery. ^†^Paired t-test. ^‡^Wilcoxon signed-rank test.

Item	Value	95% CI	Test statistic	p-value
First site of FESS, R/L	37/14			
Operative time				
First site	28 min 25 s ± 9 min 15 s	25 min 49 s-31 min 00 s	-0.5	0.5^†^
Second site	28 min 49 s ± 10 min 00 s	25 min 59 s-31 min 36 s		
Endoscopic score				
First site	1.4 ± 0.6	1.2-1.6	12	0.4^‡^
Second site	1.5 ± 0.7	1.3-1.6		

**Table 3:** Operative time and endoscopic score changes by phenotype. ECRS: eosinophilic chronic rhinosinusitis. ^†^Mann-Whitney U test.

Item	Value	95% CI	Test statistic	p-value
ECRS	23			
Non-ECRS	28			
Changes in operative time (2nd side - 1st side)				
ECRS	108.1 ± 359.4 s	-47.3 to 263.5 s	399	0.1^†^
Non-ECRS	-46.3 ± 258.2 s	-146.4 to 53.8 s		
Changes in endoscopic score (2nd side - 1st side)				
ECRS	0.1 ± 0.3	0-0.2	349.5	0.4^†^
Non-ECRS	0 ± 0.5	-0.2 to 0.2		

## Discussion

This study demonstrated that a single preoperative administration of TXA allowed bilateral full-house FESS to be performed, with comparable operative times and endoscopic scores.

TXA exerts an inhibitory effect on fibrinolysis, and its efficacy in reducing intraoperative bleeding has been demonstrated in various surgical fields beyond otolaryngology [8-10]. A recent systematic review of 23 randomized controlled trials reported that the administration of TXA reduced operative time and intraoperative bleeding during FESS, without increasing the risk of thromboembolic events, and improved surgical field visibility and surgeon satisfaction [4]. The intravenous administration of TXA reaches a peak plasma concentration approximately 15 min after injection, with a plasma half-life of approximately 2 h [11,12]. Based on this pharmacokinetic profile, we speculate that administering TXA at the start of surgery allows it to reach peak concentration during septoplasty or polypectomy, thereby exerting sufficient hemostatic effects at the beginning of FESS. These effects are presumed to have lasted throughout the bilateral full-house FESS procedure, resulting in no significant differences in operative time or surgical field scores between the sides. It has been reported that an intravenous dose of 15 mg/kg TXA is more effective than 5 mg/kg TXA [13]. In the present study, a fixed dose of 1000 mg was administered at the beginning of surgery, without adjusting the dose based on individual patient characteristics. Nevertheless, bilateral full-house FESS was performed with comparable quality. These findings suggest that repeated or continuous administration of TXA may have limited benefits in FESS. However, the potential impact of TXA administration beyond its plasma half-life was not investigated in this study and remains a subject for future research. TXA administration has been reported to be particularly effective in patients with chronic rhinosinusitis with nasal polyps (CRSwNP), a condition associated with a high risk of intraoperative bleeding [14]. In Japan, CRSwNP is widely recognized as an ECRS [15]. In the present analysis, no significant differences were observed in operative time or changes in endoscopic scores between patients with and without ECRS, indicating a similar efficacy of TXA in both groups. These findings suggest that adjusting the dosage of TXA based on phenotypic classification may not be necessary for FESS in patients with chronic rhinosinusitis.

This study had several limitations. The anatomy of the paranasal sinuses varies between individuals and between the left and right sides in the same patient. Therefore, it was not possible to standardize the difficulty of the surgical procedures completely. However, in this study, both sides of the same patient were operated on by only two surgeons using an identical surgical protocol. Given these conditions, further standardization was not feasible. Additionally, since no sample size calculation was performed, there may be limitations in the statistical analysis. However, securing eligible cases for surgery and ensuring that a limited number of surgeons perform the procedures using the same technique remained a significant challenge. It is well known that endoscopic scoring is subject to measurement bias [16]. Strictly speaking, a more accurate assessment of the time-dependent hemostatic effect of TXA could be achieved by precisely measuring the intraoperative blood loss on each side during FESS. However, these measurements are technically challenging. The primary goal of FESS is to achieve complete marsupialization of the paranasal sinuses without causing collateral damage, which was achieved in all the cases in the present study. Therefore, we believe that the impact of intraoperative bleeding on surgical progression should be adequately evaluated. In this study, a fixed dose of 1000 mg of TXA was administered to all patients regardless of their body size. However, it cannot be ruled out that different results might be obtained in cases where the surgical duration is longer. In such situations, an appropriate TXA dosage considering the patient’s body habitus should be determined.

## Conclusions

The findings of this study demonstrated that a single preoperative intravenous administration of TXA at a dose of 1000 mg enabled bilateral full-house FESS to be performed with comparable operative times and surgical field quality on both sides. Moreover, no significant differences in surgical outcomes were observed between patients with ECRS and those without, suggesting that phenotypic variations may not significantly influence the efficacy of TXA in chronic rhinosinusitis. These findings suggest that, provided the surgical procedure is completed within the approximate 2-h elimination half-life of TXA, additional or continuous dosing may not be necessary.
